# 2D-electrophoresis and multiplex immunoassay proteomic analysis of different body fluids and cellular components reveal known and novel markers for extended fasting

**DOI:** 10.1186/1755-8794-4-24

**Published:** 2011-03-25

**Authors:** Freek G Bouwman, Baukje de Roos, Isabel Rubio-Aliaga, L Katie Crosley, Susan J Duthie, Claus Mayer, Graham Horgan, Abigael C Polley, Carolin Heim, Susan LM Coort, Chris T Evelo, Francis Mulholland, Ian T Johnson, Ruan M Elliott, Hannelore Daniel, Edwin CM Mariman

**Affiliations:** 1 Department of Human Biology, NUTRIM, Maastricht University, Maastricht, the Netherlands; 2 Rowett Institute of Nutrition and Health, University of Aberdeen, Aberdeen, UK; 3 Molecular Nutrition Unit, ZIEL - Research Center for Nutrition and Food Sciences, Technische Universität München, Freising-Weihenstephan, Germany; 4 Biomathematics and Statistics Scotland, Aberdeen, UK; 5 Institute of Food Research, Norwich Research Park, Norwich, UK; 6 Department of Bioinformatics - BiGCaT, NUTRIM, Maastricht University, Maastricht, the Netherlands

## Abstract

**Background:**

Proteomic technologies applied for profiling human biofluids and blood cells are considered to reveal new biomarkers of exposure or provide insights into novel mechanisms of adaptation.

**Methods:**

Both a non-targeted (classical 2D-electrophoresis combined with mass spectrometry) as well as a targeted proteomic approach (multiplex immunoassay) were applied to investigate how fasting for 36 h, as compared to 12 h, affects the proteome of platelets, peripheral blood mononuclear cells (PBMC), plasma, urine and saliva collected from ten healthy volunteers.

**Results:**

Between-subject variability was highest in the plasma proteome and lowest in the PBMC proteome. Random Forests analysis performed on the entire dataset revealed that changes in the level of the RhoGDI2 protein in PBMC and plasma ApoA4 levels were the two most obvious biomarkers of an extended fasting. Random Forests (RF) analysis of the multiplex immunoassay data revealed leptin and MMP-3 as biomarkers for extended fasting. However, high between-subject variability may have masked the extended fasting effects in the proteome of the biofluids and blood cells.

**Conclusions:**

Identification of significantly changed proteins in biofluids and blood cells using a non-targeted approach, together with the outcome of targeted analysis revealed both known and novel markers for a 36 h fasting period, including the cellular proteins RhoGDI2 and CLIC1, and plasma proteins ApoA4, leptin and MMP-3. The PBMC proteome exhibited the lowest between-subject variability and therefore these cells appear to represent the best biosamples for biomarker discovery in human nutrigenomics.

## Background

There is an emerging demand on biomarker discovery especially in the field of nutrition with respect to weight regulation and obesity [1]. Proteomic technologies are increasingly being applied in nutrition research to reveal biomarkers that can help to demonstrate effectiveness of certain diets. Furthermore, these technologies also aid the discovery of mechanisms whereby dietary regimens influence health [2,3]. Caloric restriction or fasting is a popular and efficient way to reduce weight [4,5]. In this study we investigated how an extended fasting period from 12 h to 36 h is reflected in the proteome of several body fluids and some of their cellular components. After 36 h fasting all glycogen resources are used [6]. The proteomic applications used were classical 2D-electrophoresis combined with mass spectrometry for protein identification, as a non-targeted approach, and a multiplex-based immunoassay, as a targeted approach. Our aim was to see which type of application best detected responses to a fasting period of 36 h and which body fluid or blood cells would be most appropriate to reflect such responses.

This human trial was part of the European Nutrigenomics Organization (NuGO) Proof of Principal Study (PPS) in which various *omic *techniques were applied [7], including transcriptomics, proteomics and metabolomics [8]. This component of the PPS was designed to evaluate experimental and biological variation on the individual level in nutrigenomic experiments as a basis for future personalized nutrition concepts [9].

## Methods

### Study design and sample preparation

**Figure 1:**
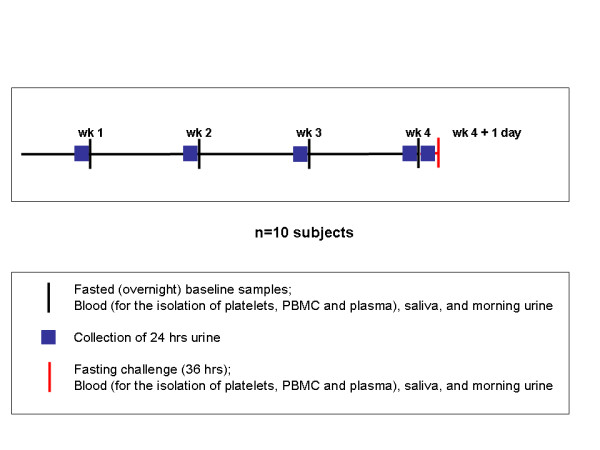
Schematic representation of the study design

The goals and design of the human PPS have been described recently [7]. Ethical permission for the study was obtained from the North of Scotland Research Ethics Services prior to the start of the study, and all volunteers gave informed consent. Ten healthy volunteers (3 males and 7 females) were enrolled at the Rowett Institute of Nutrition and Health, University of Aberdeen. The BMI of volunteers ranged from 18.5 to 39.7 kg/m2 and the age of the volunteers ranged from 25 to 56 years. The volunteers were asked to come to the Human Nutrition Unit once a week on different days each week during a four week period after an overnight fast to provide a blood sample (for the isolation of plasma, PBMC and platelets) and a saliva and 24 h urine sample. After the fourth sampling day, volunteers were fasted for an additional 24 h (total of 36 h) followed by sample collection (Figure 1). Blood samples were collected into vacutainers containing potassium EDTA anticoagulant. The isolation of platelets, PBMC, plasma, saliva and urine were performed as described by Crosley et al. [10]. Protein concentrations of platelets, PBMC, saliva and urine were assessed by the RC/DC assay (BioRad) according to the manufacturer's instructions. The protein contents of plasma samples were determined using 2-D Quant kits (GE Healthcare) according to the manufacturer's instructions. The samples were immediately aliquoted, snap frozen in liquid nitrogen, stored at -80°C and shipped to the different laboratories for analysis.

### Analysis of plasma proteins by multiplex immunoassay

The concentrations of 89 proteins were measured by Rules-Based Medicine (RBM) in all plasma samples by a multiplex immunoassay (HumanMAP Version 1.6, Rules-Based Medicine, Inc., Austin TX, USA).

### 2D-electrophoresis

The platelets and saliva were analyzed in laboratory 1, PBMC and urine in laboratory 2 and depleted plasma in laboratory 3. Each of the three laboratories ran one gel per sampling time point, the 4 overnight fasting time points were considered as replicates. 2D-electrophoresis was performed as described previously [11]. Briefly, for the blood related samples 200-250 μg protein was loaded onto a 24 cm 4-7 linear IPG strip for separation in the first dimension. Prior to IEF the plasma samples were depleted of Albumin and IgG using the Albumin and IgG Removal Kit (GE Healthcare) according to the manufacturer's instructions. The second dimension separation was on a standard 12% SDS-PAGE for platelets and PBMC, the second dimension of the depleted plasma was separated on a 1 mm thick 10% Duracryl gel [11]. For the saliva samples, 25 μg protein was loaded onto an 11 cm 4-7 linear IPG strip for separation in the first dimension. The second dimension separation was carried out on a 12% SDS-PAGE. For the urine samples 300 μg protein was loaded onto an 18 cm pH 4-7 linear IPG strip. The gels with urine proteins were stained with Coomassie. Gels with proteins obtained from other biofluids and blood cells were stained with Flamingo fluorescent stain (Biorad) according to the manufacturer's instructions. Spots were identified and analyzed using the PDQuest v8.0 software (Biorad) for the platelets and saliva; Delta2D v3.6 (Decodon) for the PBMC and urine and Progenesis SameSpots v3.1 (Nonlinear Dynamics) for depleted plasma. Background subtraction and normalization were automatically carried out by the software programs.

### Protein identification

The selected protein spots were excised from the 2D gels and processed for protein identification in the same laboratory where the gels were run. Generation of tryptic digest from the protein by in-gel digestion was performed as described previously [11,12].

#### MALDI-TOF/TOF mass spectrometry

For MS/MS analysis of the spots from platelets and saliva (laboratory 1), 1.0 μl of the peptide mixture and 1.0 μl matrix solution (2.5 mg/ml CHCA in 50% acetonitrile/0.1% trifluoroacetic acid; TFA) were spotted on a 384-well MALDI target plate. Data were collected with a MALDI-TOF/TOF mass spectrometer (4800 MALDI-TOF/TOF Proteomics Analyzer, Applied Biosystems). A default calibration was applied using a six-component peptide standard spotted onto 13 different positions of the MALDI target plate for MS, and Glu-Fibrinopeptide B (m/z 1570,68) fragmentation for MS/MS. Typically, 1000 shots were combined for obtaining MS spectra and for MS/MS spectra 2000 shots were combined. The GPS Explorer v3.6 software (Applied Biosystems) was employed to generate peak lists and automated MASCOT searches against the Swiss-Prot protein database (Swiss-Prot release 56.5; 402 482 sequences) for protein identification. One miss-cleavage was tolerated; carbamidomethylation was set as a fixed modification and oxidation of methionine as an optional modification. The protein charge was set at 1+. The precursor tolerance was set to 100 ppm and the MS/MS tolerance was set at 0.2 Da. No restrictions were made on the protein mass. Protein hits with a MASCOT protein score greater than 56 (p < 0.05) and a GPS Explorer protein score confidence index >95% were validated as confirmed identifications [13].

The identity of the plasma, PBMC and urine spots (laboratory 2 and 3) was determined by peptide fingerprinting using an Ultraflex MALDI-TOF/TOF mass spectrometer (Bruker Daltonics Ltd.). A 200 Hz nitrogen laser was used to desorb/ionize the matrix/analyte material, and ions were detected in positive ion reflectron mode. All spectra were acquired automatically using the Bruker fuzzy logic algorithm (FlexControl 3.0, Bruker) and a Biotools 3.0 search routine. The resulting mass data was interrogated using an offline version of the MASCOT search engine (Matrix Science) using the SPtrEMBL database with the following search criteria: allowance of 0 or 1 missed cleavages; peptide mass tolerance of 50 ppm; trypsin as digestion enzyme; carbamidomethyl modification of cysteine; methionine oxidation as partial modification; and charged state as MH+ [14].

### Statistical analysis

Exploratory analysis of the data indicated that a log-transformation was advisable for most data sets. For reasons of consistency all data sets were analyzed on a log_2_-scale and back transformed to report fold changes. For the extended fasting effect analysis, a linear model including time point (5 levels) and subject (10 levels) as factors was fitted to the 50 data points for each variable. The extended fasting effect was then defined as a contrast that compared time point 5 (36 hours fasting) with the average of the 4 baseline measurements (overnight fasting). The 4 baseline time points were used to determine the between-subject effect, again using a linear model with subject as the only factor. P-values were adjusted for multiple testing within each data set by using the Benjamini-Hochberg method.

Spots were selected for protein identification if their adjusted p-value fell below 0.2 allowing a false discovery rate (FDR) of 20%. Linear model analysis and P-value adjustment were performed using the R package v2.10.1. In order not to miss any interesting spots we also performed additional analyses on the original scale and also selected proteins with an adjusted P below 0.2 on this scale. In this way we identified 10 proteins from the 2D-electrophoresis and 39 proteins for the multiplex immunoassay which were significantly changed upon the extended fasting. The data were imported into GeneMaths v2.11 for discriminant principal component analysis and cluster analysis with Pearson correlation. We used the R package v2.10.1 for random forests (RF) analysis, RF package 4.5-34 [15]. RF analysis was applied to normalized data. The number of trees was set to 500 and the number of variables randomly sampled as candidates at each split was set to 9. Ten cycles were calculated and averaged. These average mean decreased Gini results were plotted. The Gini importance indicates the importance of the variable in the classification. Cytoscape v2.7 with the MiMI plug-in 3.0.1 was used to create network associations. All proteomic data (2D and RBM) where used for creating the network. The protein-protein interaction network was created using all available databases present in MiMI, among which are IntAct, BIND and KEGG [16].

## Results

In the proteome of most biofluids and blood cells an effect of an extended fasting period from 12 h to 36 h could be observed. However, the number of proteins that changed as a result of the extended fasting was relatively small compared to the large number of proteins that differed significantly between subjects. Of the 2971 detected spots/proteins in all data sets, 153 spots/proteins were changed due to extended fasting (5.1%), whereas 914 mostly other spots/proteins exhibited significant between-subject differences (30.8%). In plasma, for example, extended fasting resulted in a significant alteration of only 1.3% of the protein spots, whereas 69.1% of the spots differed significantly between subjects, when using 2D-electrophoresis. Blood cells, on the other hand, showed a remarkably lower number of proteins that exhibited significant between-subject differences (0.3% and 0.4% of the proteins in platelets and PBMC, respectively). Moreover, PBMC showed the highest number of proteins that changed significantly when volunteers extended fasting from 12 h to 36 h (9.1%). With the targeted approach using RBM, 39 plasma proteins (43.8%) changed significantly due to extended fasting, and 78 proteins (87.6%) differed significantly between subjects (Table 1).

**Table 1:** Number of protein spots/proteins that change during fasting challenge

	Fasting effect	Subject effect	Total detected spots/proteins
*Non-targeted approach*			
Plasma proteomics	13	703	1018
PBMC proteomics	83	4	910
Platelet proteomics	0	2	604
Saliva proteomics	1	69	208
Urine proteomics	3	58	142
*Targeted approach*			
Rules Based Medicine	39	78	89

A fasting and a between-subject effect was observed.

**Figure 2:**
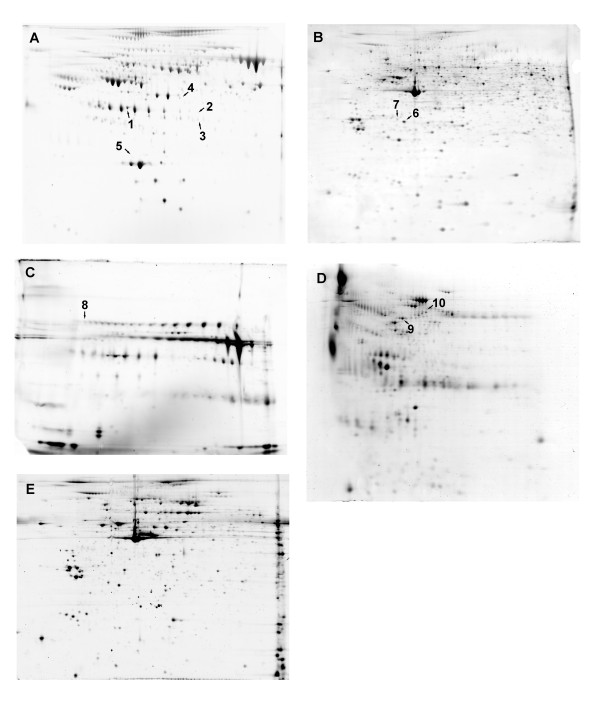
**Identified proteins marked on 2D gels representing plasma (A), PBMC (B), saliva (C), urine (D) and platelets (E)**. The numbers refer to those used in Table 2.

Using the 2D-electrophoresis approach we identified proteins from spots with a relatively highly significant adjusted p-value as a result of extended fasting: 5 spots in plasma, 2 spots in PBMC, 1 spot in saliva and 2 spots in urine (see Table 2 and Figure 2). Using the targeted approach, we were able to identify 39 proteins that changed significantly upon 36 h fasting compared to overnight fasting (Table 3). The chloride intracellular channel protein 1 (CLIC1) in PBMC exhibited a 5.3-fold increase upon the fasting challenge. This was the most pronounced change amongst all proteins (Table 2). C reactive protein (CRP) was detected as increasing in both the plasma 2D proteome and the RBM plasma data set induced by extended fasting (Table 2 and 3).

**Table 2:** Protein identifications by MS/MS analysis of biofluid and blood cell proteins which were significantly increased or decreased upon extended fasting (36 h), as compared with overnight fasting (12 h)

Sample type	Spot	Accession #	Protein identification	Fold- change	p-value	Adjusted p-value	Mascot score	Sequence Coverage %	Matched peptides
Plasma	1	P06727	Apolipoprotein A-IV (ApoA4)	-1.80	<0.001	<0.001	426	69	34
	2	P00738	Haptoglobin B chain	-1.15	<0.001	0.009	87	32	8
	3	P00738	Haptoglobin B chain fragment	-1.14	<0.001	0.006	139	33	17
	4	P36955	Pigment epithelium-derived factor	-1.09	<0.001	0.061	113	35	16
	5	P02741	C reactive protein (CRP)	1.42	<0.001	0.061	74	17	6
PBMC	6	P52566	Rho GDP-dissociation inhibitor 2 (RhoGDI2)	-2.78	<0.001	<0.001	63	23	4
	7	O00299	Chloride intracellular channel protein 1 (CLIC1)	5.30	<0.001	<0.001	73	23	4
Saliva	8	P01833	Polymeric-immunoglobulin receptor (PIGR)	-3.16	<0.001	0.030	161	15	10
Urine	9	P02768	Serum albumin precursor	1.83	0.004	0.185	162	45	22
	10	P02768	Serum albumin precursor	2.02	0.001	0.056	152	32	16

**Table 3:** Plasma proteins of which absolute levels significantly changed due to extended fasting (36 h) compared with overnight fasting (12 h), as assessed using the multiplex immunoassay (RBM)

Protein	Accession #	Fold-change	p-value	Adjusted p-value
IGF-1	P01343	-2.72	<0.001	<0.001
Leptin	P41159	-2.59	<0.001	<0.001
Erythropoietin	P01588	-1.56	0.080	0.187
IgE	n.a.	-1.54	0.002	0.013
IL-8	P10145	-1.51	<0.001	0.001
MMP-3	P08254	-1.48	<0.001	<0.001
Thyroid Stimulating Hormone	P01222	-1.47	<0.001	<0.001
Insulin	P01308	-1.29	<0.001	0.001
Carcinoembryonic Antigen	P06731	-1.29	0.037	0.116
IL-1beta	P01584	-1.19	<0.001	<0.001
PAI-1	P05121	-1.19	0.002	0.013
Apolipoprotein A-1 (ApoA1)	P02647	-1.18	0.006	0.025
MCP-1	P13500	-1.17	<0.001	0.001
Eotaxin	P51671	-1.16	0.001	0.005
VEGF	P15692	-1.14	<0.001	<0.001
IL-16	Q14005	-1.13	<0.001	0.002
EGF	P01133	-1.12	0.085	0.190
IL-18	Q14116	-1.12	0.013	0.048
Alpha-Fetoprotein	P02771	-1.12	<0.001	<0.001
Fatty Acid Binding Protein	P05413	-1.07	0.006	0.025
IgA	n.a.	-1.07	0.043	0.116
Factor VII (F7)	P08709	-1.06	0.061	0.157
Creatine Kinase-MB	P06732	-1.04	0.075	0.181
IL-10	P22301	-1.04	0.041	0.116
Glutathione S-Transferase	P09211	-1.04	0.010	0.041
Tissue Factor (TF)	P13726	-1.03	0.004	0.019
Prostatic Acid Phosphatase	P15309	-1.03	0.018	0.067
Alpha-2 Macroglobulin (A2M)	P01023	1.02	0.021	0.072
Complement 3	P01024	1.02	0.028	0.092
TIMP-1	P01033	1.02	0.005	0.025
IL-12p70	P29459	1.03	0.038	0.116
VCAM-1	P19320	1.05	0.011	0.044
Serum Amyloid P (SAP)	P02743	1.07	0.072	0.179
Lipoprotein (a)	P08519	1.09	0.041	0.116
MIP-1beta	P13236	1.09	0.048	0.127
TNF-alpha	P01375	1.10	0.042	0.116
EN-RAGE	P80511	1.17	0.085	0.190
Ferritin	P02794	1.27	<0.001	0.001
C Reactive Protein (CRP)	P02741	1.55	<0.001	0.002

n.a. = no accession #

**Figure 3:**
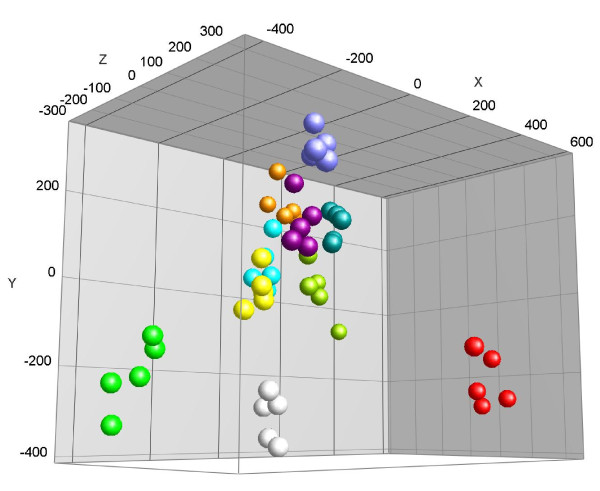
**PCA analysis of data obtained from the multiplex immunoassay (RBM)**. Each color represents a different subject

Principal component analysis applied to the multiplex immunoassay (RBM) data set revealed that each of the subjects could be identified based on levels of 89 plasma proteins (Figure 3). It appears that such data can be used to provide a metabolic fingerprint of the individual volunteers participating in this intervention study. However, this demonstrates that the between-subject effects are larger than that of the fasting effect.

**Figure 4:**
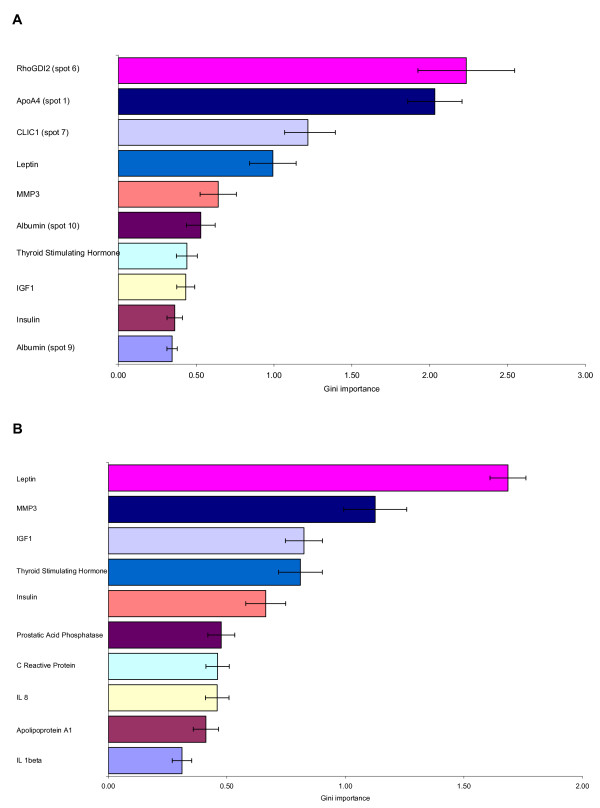
**Biomarkers of extended fasting effect calculated by Random Forrest analysis**. Panel A: results obtained from analysis of all proteomic data (2D and RBM). Panel B: results obtained from targeted proteomic analysis using multiplex immunoassay (RBM). The spot numbers between brackets refer to those used in Table 2. Proteins without numbers between brackets refer to those used in Table 3.

RF analysis applied to the full data set from non-targeted and targeted proteomics applications, revealed that the Rho GDP-dissociation inhibitor 2 (RhoGDI2) in PBMC and the plasma Apolipoprotein A-IV (ApoA4) appeared the most significant biomarkers associated with extended fasting (Figure 4a). RF analysis of the targeted proteomics data set revealed leptin and matrix metalloproteinase-3 (MMP-3) as biomarkers of extended fasting (Figure 4b).

**Figure 5:**
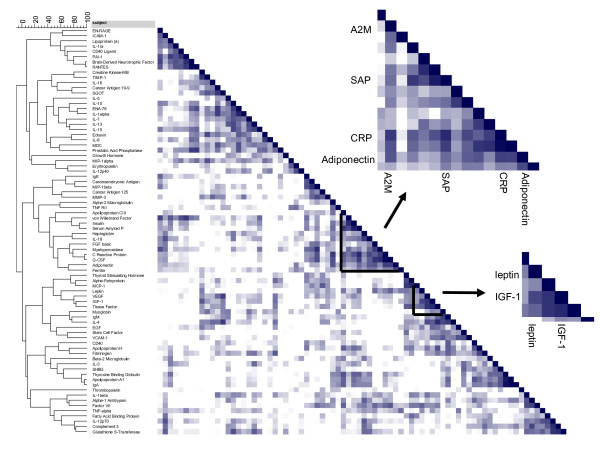
**Correlation score on cluster analysis of Heat map of the Pearsons correlations comparing fold-changes in plasma proteins after a 36 h fasting versus overnight fasting**.

**Figure 6:**
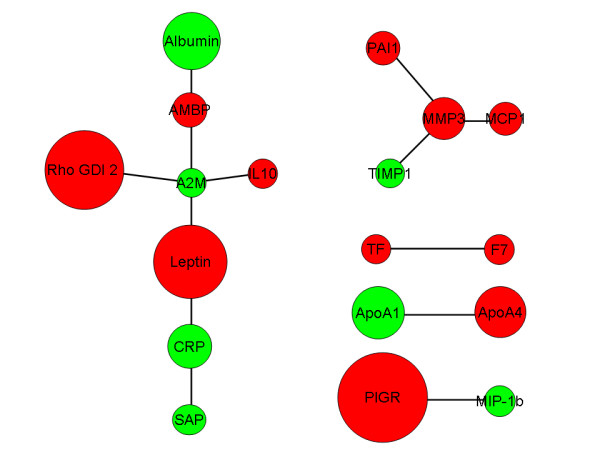
**Protein-protein interaction networking based on the outcome of the total dataset**. Red represents a decrease and green an increase in protein levels. The size of the circle represents the fold change levels. The short protein names shown in the network refer to Table 2 and 3. Alpha-1-microglobulin (AMBP) was down-regulated and ApoA1 was up-regulated in urine, but not significantly (data not shown).

Cluster analysis with Pearson correlations between the fold-change in RBM plasma proteins after extended fasting versus overnight fasting is shown in Figure 5. Leptin and Insulin-like growth factor I (IGF-1) are correlated and closely clustered to each other. Both proteins are down-regulated after extended fasting (Table 3). CRP is correlated to changes in alpha-2-macroglobulin, serum amyloid P and adiponectin (Figure 5). The close association between CRP, serum amyloid P and alpha-2-macroglobulin is confirmed in the protein-protein interaction network shown in Figure 6. In addition, leptin changes appear directly linked to changes in CRP and alpha-2-macroglobulin, whereas MMP-3 appears directly linked to alterations in the tissue inhibitor of metalloproteinase-1 (TIMP-1), monocyte chemotactic protein-1 (MCP-1) and plasminogen activator inhibitor-1 (PAI-1).

## Discussion

This study, using both non-targeted as well as targeted proteomic approaches applied to biofluids and blood cells to elucidate changes in their proteomes induced by extended fasting, revealed substantial between-subject variability which may mask the true biological effect of fasting on the proteome. This suggests that any nutrigenomic experiment require a sufficient number of replicates and matched samples to identify the usually rather subtle changes that dietary maneuvers may impose. Levels of between-subject variability were higher in all biofluids samples as compared to the cellular proteomes. In this study, PBMC exhibited the largest number of significant changes in protein levels following the extended fast. RF analysis on the entire data set revealed that in PBMC the RhoGDI2 and plasma ApoA4 proteins were the most significant biomarkers for extended fasting. RF analysis of the multiplex immunoassay data set revealed leptin and MMP-3 as potential biomarkers for extended fasting.

It should be noted that the between-subject variation may in part be due to the heterogeneous composition of our study cohort with regard to various parameters, including gender and BMI. Consequently, this type of variability can be reduced by selecting a more homogeneous study population. We observed markedly less inter-individual difference in the PBMC proteome than we observed previously in the PBMC transcriptome of PBMC from human volunteers who were participating in a study with a similar baseline design [17]. In our study 0.4% of the PBMC proteins detected differed significantly between individuals compared with 39% of PBMC transcripts [17].

Most interesting biomarkers that are involved in metabolic pathways, as well as those related to inflammation and oxidative stress, are present only in very low concentrations in biosamples. In these cases, their low abundance prevents detection by classical proteomic techniques such as 2D-electrophoresis [18]. Multiplex immunoassays are therefore an interesting alternative because they are more sensitive compared to 2D-electrophoresis [19]. Indeed, a wide range of plasma protein changes caused by extended fasting were detected using the targeted multiplex immunoassay method. However, a big advantage of 2D-electrophoresis is that it can visualize isoforms and possible protein modifications, whereas with an antibody-based approach this depends on the availability of isoform-specific antibodies.

CLIC1 in PBMC was the protein that exhibited the largest fold-change as a result of the extended fasting. In general, chloride channels play important roles in the regulation of cellular excitability, transepithelial transport, cell volume regulation, and acidification of intracellular organelles [20]. CLIC1 is responsible for stabilization of the membrane potential, which could be influenced by the nutrient status in PBMC. Moreover CLIC1 seems to be associated with cellular stress response mechanisms and starvation may be interpreted as a metabolic stress condition [21].

Besides CLIC1, levels of RhoGDI2 and ApoA4 were the best indicators of the extended fasting state based on analysis of all the proteomic data. Insulin has the ability to relocate RhoGDI2 to the membrane [22]. Since plasma glucose and insulin levels are low during fasting, the relative amount of RhoGDI2 in the cytosol of PBMC might be predicted to increase during extended fasting. Since by 2D preferentially cytosolic proteins are detected, RhoGDI2 is expected to rise in spot intensity. However, a decrease was noticed in our experiment indicating that cytosolic RhoGDI2 concentration is influenced by other factors like posttranslational modification or turn-over rate.

ApoA4 is a major component of chylomicrons and is synthesized by the small intestine. It is proposed to represent a circulating satiety signal [23] and after fasting, therefore, may decrease its levels [24].

IGF-1, leptin and CRP were the proteins with the biggest negative and positive fold change upon the fasting challenge in the RBM data. Circulating IGF-1 levels are associated with dietary protein intake [25,26]. This could explain why after an extended fast without protein intake, IGF-1 is decreased [27]. The decrease of leptin is as expected because it is known to be a positive marker for fasting or weight loss [28-30]. Chan et al. [31] have shown that leptin and IGF-1 levels decrease after a 72 h fasting. Our correlation analysis clustered IGF-1 and leptin suggesting that adipose tissue, as the main source of leptin, and liver, as the origin of circulating IGF-1, undergo metabolic adaptation via a closely linked mechanism. CRP is an acute phase protein and its elevation after this short term fasting might therefore be expected, although after long term fasting or caloric restriction CRP is usually down-regulated [32]. RF analysis applied to the RBM data revealed leptin and MMP-3 as the most obvious biomarkers for a fasting challenge. No direct interaction between leptin and MMP-3 has been reported so far. A possible mechanism could be the inverse correlation between leptin and adiponectin levels [19,33] since the leptin/adiponectin ration changes during extended fasting. Adiponectin by itself has the ability to increase TIMP1, which is able to inhibit the activity of MMPs [34,35]. However, adiponectin plasma levels did not change significantly (p = 0.45) upon the extended fasting.

Most interestingly, the correlation analysis clustered CRP closely together with adiponectin, serum amyloid P and alpha-2-macroglobulin. Except for adiponectin, a networking interaction analysis using MiMI, places those proteins together with leptin and RhoGDI2. An interaction between leptin and CRP, which are positively correlated in normal weight, overweight and obese subjects [36], was described by Chen et al [37]. Alpha-2-microglobulin is described as a leptin binding protein [38]. This interaction may be involved in the clearance of leptin by the alpha-2-microglobulin receptor.

Another observation from the networking analysis was an interaction between PAI-1, MMP-3, MCP1 and TIMP1. As mentioned above, TIMP1 inhibits the activity of MMPs [34,35], whereas both PAI-1 and MCP1 are inactivated through proteolysis mediated by MMP-3 [39,40]. This interaction between MMP-3 and MCP-1 may be the reason that MMP-3 is ranked relatively high by the RF analysis. An inhibition or decrease of PAI-1 leads to a reduction of fat depots and adipocyte volume [41]. Several studies have shown that PAI-1 is increased in adipose tissue and plasma in obese humans and that plasma PAI-1 concentrations decrease after weight loss or fasting [42,43], which suggests a role for this proteinase inhibitor in the development and maintenance of obesity. Jensen et al. [44] reported that a low glycemic index diet in overweight adults could be beneficial in regulating fasting concentrations of the cardiovascular disease risk factor PAI-1 showing that PAI-1 concentrations are sensitive to the nutritional status. PAI-1, TIMPs and MMPs play a role in extracellular matrix remodeling [41,45,46]. Interestingly, a correlation has been described between CRP and PAI-1 [47,48], linking both interaction networks.

## Conclusions

Both non-targeted and targeted proteomic analysis on a range of human body fluid samples and selected blood cells examining the effect of an extended fasting revealed that there is a considerable between-subject effect with the lowest variability in the PBMC proteome. These cells may therefore be considered as a prime sample source in further nutrigenomic studies when proteomic technologies are applied for biomarker discovery. The antibody based analysis is superior when plasma proteins are analyzed and reveals a distinct metabolic fingerprint that identifies the individuals. The protein panel that characterizes extended fasting by the most significant changes comprises known entities such as leptin, ApoA4 and IGF-1, but also a variety of novel markers including RhoGDI2, CLIC1 and MMP-3. These candidate biomarkers could be of interest in future nutrition studies.

## Authors' contributions

FB designed and performed the experiments, analyzed the data and wrote the manuscript. BR conceived, designed and coordinated the study, analyzed the data. IRA designed the study, performed the experiments and analyzed the data. KC designed the study and collected the samples. SD designed and coordinated the study. CM collected the raw data and performed the statistic analysis. GH performed the statistic analysis. AP designed and performed the experiments. CH designed and performed the experiments. SC participated in the data analysis. CE participated in the data analysis. FM designed the study and performed the experiments. IJ designed the study and performed the experiments. RE designed and coordinated the study, performed the experiments and analyzed the data. HD designed and coordinated the study. EM designed and coordinated the study and participated in manuscript writing. All authors read and approved the final manuscript.

## Competing interests

The authors declare that they have no competing interests.

## Pre-publication history

The pre-publication history for this paper can be accessed here:

http://www.biomedcentral.com/1755-8794/4/24/prepub
